# Soil Remediation Practices for Hydrocarbon and Heavy Metal Reclamation in Mining Polluted Soils

**DOI:** 10.1155/2018/5130430

**Published:** 2018-11-18

**Authors:** Nigel Makombe, Reginald Dennis Gwisai

**Affiliations:** Department of Environmental Science, Bindura University of Science Education, Zimbabwe

## Abstract

The study assessed the pollution, biodegradation rates, and phytoaccumulation of total petroleum hydrocarbons (TPHs), Lead (Pb), Cadmium (Cd), and Arsenic (As) in soils in the wet and dry seasons and compared them with set standards. Ten samples of 1kg each were randomly collected and mixed for each site that is the upgradient control site (10m^2^ strata design) and the downgradient contaminated site (16m^2^ strata design) to make a composite sample for each site. Three representative samples were collected and replicated for four months on both sites. Pot experiments were run with the same concentration levels of TPHs, Pb, As, and Cd. Each pot experiment was also replicated four times for tobacco compost, chicken droppings, Brassica juncea, and the control. Inductive Coupled Spectrometry, SPSS, ANOVA, t-test, normality, and post hoc tests were carried out for analysis. TPHs, Pb, As, and Cd concentrations were significantly higher (p<0.05) than the limits* (MHSPEN and USEPA)*. TPHs and heavy metals had the highest concentration levels in the soil at the selected site in the following order: TPHs>Pb>Cd>As. Bioremediation in a controlled experiment revealed that Chicken Droppings and Brassica juncea were effective in reclaiming TPHs, As, and Cd while Tobacco Compost was effective in reclaiming Pb. The highest mean concentrations of Pb, As, and Cd were found in Brassica juncea in the following increasing order: roots, stem, and leaves, respectively.

## 1. Introduction

The use of bioremediation as a cleanup strategy for contaminated environments has increased due to its viability and cost-effectiveness [1–3]. Previous studies have shown that bioremediation uses biological agents such as fungi, bacteria, and green plants (phytoremediation) to remove, mineralize or neutralize hazardous substances in soil [1, 2, 4]. Bioremediation is divided into two types which are natural attenuation and engineered bioremediation to allow environmental conservation [4, 5]. Engineered bioremediation has* in situ *remediation which entails remediation at the source of pollution and* ex situ* remediation which involves excavating soil from the point of pollution [4, 5]. In developing countries waste management has become a major problem that is coming with health risks due to heavy metals and other types of biohazard pollution [6, 7]. In Zimbabwe there are widespread environmental problems regarding heavy metal pollution and petroleum hydrocarbons due to poor waste handling, wastewater disposal, and other anthropogenic activities [1, 3, 8, 9]. Due to the ever increasing waste disposal rates on the land and water, heavy metals have become a major health risk due to ground and surface water pollution [4, 6, 10, 11]. Most of the agricultural land is polluted due to irrigation with waste water and urban soils are contaminated due to improper waste water disposal and industrial activities [3, 6, 12]. Previous studies have revealed that wastewater use for irrigation and waste disposal on the soil results in heavy metal accumulation in soils and bioaccumulation in plants beyond maximum permissible limits for both humans and livestock consumption [3, 13, 14]. Therefore soil contamination challenges in Zimbabwe need solutions such as bioremediation and chemical and physical methods of treatment [1, 4, 6].

Chemical and physical mechanisms of treating contaminated soil involve excavation, separation, extraction, electro kinesis, washing, oxidation, and reduction [1]. However, high costs and major disruptions associated with these treatment methods have limited their adoption [15]. On the other hand, bioremediation is preferred to other treatment methods due to its cost-effectiveness and efficiency in contaminant removal as it leaves the soil intact without any disruptions to the ecosystem and soil structure [1, 2, 4, 8]. Bioremediation processes occur when biological degradation takes place in the cells of microorganisms which absorb the hazardous substance leading to specific enzymatic metabolism [5]. Petroleum hydrocarbons are used by microbes as a source of energy and nutrients and they are neutralized or decomposed to form naphthenic acids, alcohols, phenols, hydroperoxides, carbonyl compounds, esters, and eventually carbon dioxide and water [1]. It has to be noted that very few bacteria strains grow in petroleum hydrocarbon contaminated environments [1, 5].

Petroleum hydrocarbons,* As, Pb,* and* Cd* are mineralized and degraded aerobically and/or anaerobically by microbes into nonhazardous substances which are integrated into the natural biochemical processes [1, 5]. The focus on aerobic decomposition of total petroleum hydrocarbons and mineralization has been emphatic because it is a rapid process, is easy to do, and has been proved in real environments for several studies. Selected plant species appear to mineralize heavy metals and decompose petroleum hydrocarbons by using their root system to absorb. Plants take up heavy metals available or soluble metals from the soil and decontaminate through phytoextraction, phytovolatilization, and phytostabilization [4, 6]. Plants used to remediate polluted areas should have characteristics that include hyper-accumulation [4, 5].

*Brassica juncea *has been shown to grow fast and is also a hyper-accumulator that tolerates heavy metal contamination and petroleum hydrocarbons contamination [2]. Also,* Brassica juncea* has the ability to reduce contamination of the food chain through hyper-accumulation since it has high amounts of thiocyanates which make them unpalatable to animals [5]. In addition, remediation solutions to soil contamination should come from the point of pollution for cost-effectiveness [2, 4]. This is in sync with the use of chicken droppings and tobacco compost waste as they are readily available while their potential is due to the presence of* Staphylococcus, Streptococcus, Corynebacterium, Pseudomonas, and Acinetobacter species* which have been identified to be tolerant to heavy metals and petroleum hydrocarbons [12, 16]. Many studies reveal the presence of these bacteria in chicken droppings and tobacco compost [5]. However, the composts generally are yet to be tested for their efficiency in mineralizing heavy metals and decomposing total petroleum hydrocarbons concurrently. Therefore, it is indispensable to test their potential in addressing the current problem faced by organizations in a cost-effective manner that is easy to control [4, 6].

## 2. Methodology

**Figure 1:**
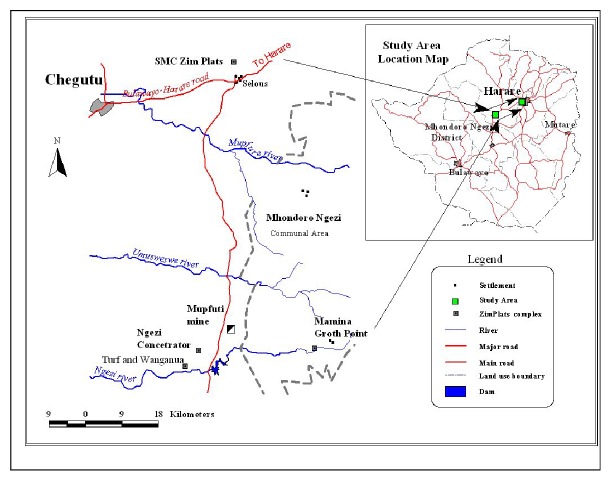
Location of the Study Area (Source: Nyamande, 2015).

A complete randomised design was used for the study. Soil was collected randomly from a 160m^2^ area where the contaminated waste was dumped. The selected area was divided into 10 strata that were 16m^2^ in size to homogenously collect samples. Ten samples of 1 kilogramme each were taken randomly from each stratum at a depth of 0-20cm to make one composite sample and 3 representative samples were then picked (Figure 1). This was replicated for 4 months from March, April, July, and August 2015 to understand the baseline trend of soil contamination with seasonal variations. Upgradient, a control site in pristine form with limited anthropogenic influence was determined 300 metres from the contaminated site which was approximately 100m^2^. Ten soil samples were taken from the control site to make one composite sample in which 3 representative samples were picked and this was repeated for 4 months the same way it was done for the selected site. Soils with the same TPHs, Lead, Arsenic, and Cadmium concentration levels were placed in the pots. Each pot experiment was replicated 4 times meaning 4 pots for tobacco compost, chicken droppings,* Brassica juncea, *and the control experiment. Soxhlet extraction was used for all the samples that were analysed in the study. The method that was used for the analysis of* As*,* Pb,* and* Cd* was acid digestion (Method 3050B). The APHA standard methods 1995 were followed for Quality Assurance purposes. Inductive Coupled Spectrometry was used for analysis of* Cd, As,* and* Pb*. Analysis was done using SPSS version 20.0. All the data was tested for normality using QQ-plots. A one sample* t*-test was used to analyse data comparing TPHs against (MHSPEN standard, 2000) and* Pb*,* As,* and* Cd* against USEPA standards, 1991. ANOVA and Post* hoc* were used to detect differences in concentrations and to compare the accumulation of pollutants in the roots, stem, and leaves.

## 3. Results and Discussions

### 3.1. TPHs, Lead, Arsenic, and Cadmium Concentration in Soil

There was a statistically significant difference in selected and control sites for all test parameters. On the selected site there were high concentration levels for TPHs,* Pb*,* As,* and* Cd *as compared to the control site (Table 1). This appears to be similar to previous studies that have shown high levels of heavy metal pollution in contaminated sites [3, 10, 16].

### 3.2. Contamination Factors for the Selected Site

The contamination factor CF<1 refers to low concentration; 1≤CF<3 means moderate contamination; 3≤CF<6 indicates considerable contamination; and CF>6 indicates very high contamination [10].  Table 2 indicates very high pollution levels at the selected sites. Furthermore, previous studies in South Africa, Nigeria, Spain, India, Iran, Vietnam, and China show a similar pattern of heavy metal pollution in soils closer to mining areas [1, 10].

**Figure 2:**
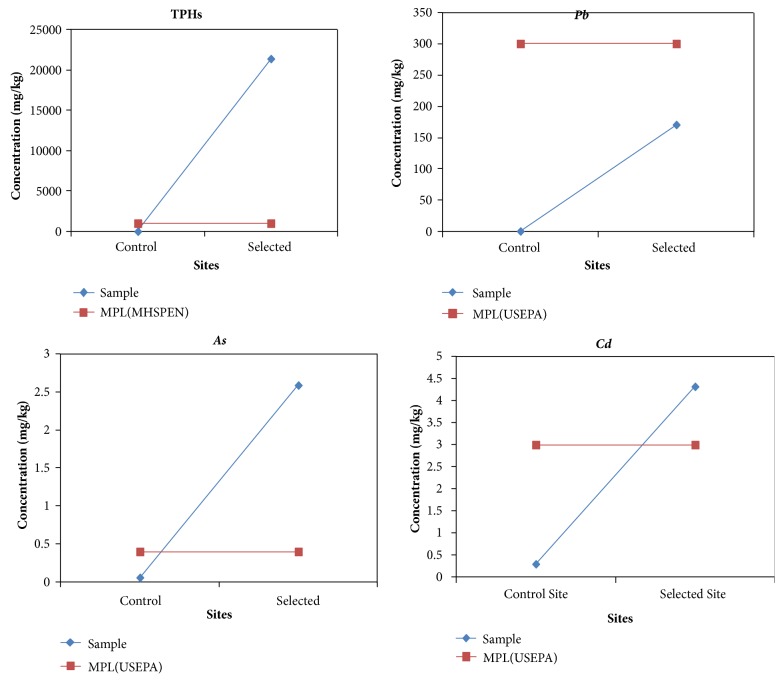
Comparison of TPHs and heavy metal concentration levels with standards MPL.

Figure 2 shows the concentration of TPHs,* Pb*,* As,* and* Cd* against standards MPL. Figure 2 shows that TPHs on the selected site was above the MHSPEN, 2000 standard MPL while the control site was below the standard. On the selected site all the test parameters were above the standards MPL except for* Pb, *showing a relatively similar pattern with past studies [10, 16].

The selected site from the results shown by Tables 1 and 2 and Figure 2 showed that there was a very high concentration of TPHs,* Pb*,* As,* and* Cd* in the soil which was above the MHSPEN, 2000, and USEPA, 1991, standards. Furthermore, the pollutant levels were considerably higher than the surrounding environment as contrasted by the concentration levels in the control site. The results on the selected site indicate major anthropogenic activities that are causing massive pollution on the soil as also observed in previous studies [1, 3, 4]. TPHs and heavy metals had the highest concentration levels in the soil at the selected site in the following order: TPHs>*Pb*>*Cd*>*As. *Naser et al. [17] mentioned that limited attention has been given to the likelihood of pollution by other heavy metals which may originate from automobiles, tyre wear (tyre material ~ 20 – 90* mg/kg*), and motor oils. This is a reflection of the cause of pollution at the site due to oils and waste from trackless mobile equipment servicing and maintenance. The contamination factor greater than 6 indicates very high contamination as observed by Aluko et al. [10]; thus the selected area was heavily contaminated. The implications of high contamination levels of heavy metals and TPHs such as alkaline, benzene, methyl benzene, and PAH in the soil as highlighted by previous studies [18, 19], cause environmental damage especially to our living ecosystem [4, 6]. The oil and heavy metal components are toxic to human and wildlife through water and food contamination and are classified as carcinogens [1, 11, 13].

TPHs,* Pb*,* As,* and* Cd *were analysed from 0 to 20cm depth level and that depth level showed that there was massive soil contamination; thus vertical movement and ground flow of contaminants can lead to ground water and surface water contamination [3, 8, 9]. Observations from previous studies show that metal soil interaction is such that when metals are introduced at soil surface, downward movement does not occur to an extent unless the metal retention capacity of the soil is overloaded [11]. Thus, with the results of the contamination levels shown for TPHs,* Pb*,* As,* and* Cd *the soil will be eventually overloaded and contaminate groundwater resulting in massive water pollution and negative implications to human health [1]. This affects the polluted sites and the surrounding communities that depend on ground and surface water for consumption as observed by previous studies [1, 10, 11, 20].

### 3.3. Wet and Dry Season Concentration Variations

Table 3 shows that from March to August the contamination level for TPHs,* Pb*,* As,* and* Cd *was increasing. There was a statistically significant difference for concentration levels in all test parameters in the wet season (March and April) as compared to the dry season (July and August). The dry season (August) recorded the highest concentration levels for TPHs,* Pb*,* As,* and* Cd*.

TPHs are less dense than water; hence they are more likely to be carried away during the wet season than in the dry season. Heavy metal ions are dense; thus they are more likely to move downwards vertically than horizontally through overland flow [4, 18]. In the wet season there might be downward movement beyond the sampling depth of 0-20cm. This explains why the concentration of pollutants in the soil has seasonal variation as observed by previous studies on heavy metals and hydrocarbon pollution [3, 4, 21]. The concentration was low in wet season as compared to dry season; this was because water was a dispersion agent. This seasonal variation was attributed to vertical migration of water carrying pollutants and overland flow of contaminants with water. The wet season also accelerated pollution of ground water and surface water bodies causing massive pollution which was linked to negative implications to the ecosystem and human health [6, 10].

### 3.4. Mineralization and Degradation of TPHs,* Pb*,* As,* and* Cd*

Table 4 shows that chicken droppings were the treatment with the highest remediation rate for TPHs,* As,* and* Cd*. Tobacco compost was the highest in remediating* Pb*. There was limited remediation on the control experiment. There was a statistically significant difference on the bioremediation of TPHs,* Pb*,* As,* and* Cd* rates among* Brassica juncea*, tobacco compost, chicken droppings, and the control experiment.

*Brassica juncea* plant was able to degrade TPHs,* Pb*,* As,* and* Cd* at a considerably higher rate as compared to tobacco compost probably because the plant is a hyper-accumulator as observed in previous studies [4, 14, 18]. Microorganisms that were attached to the rhizosphere can also degrade and mineralize* Pb*,* Cd*,* As,* and TPHs in soil as shown by the results in Table 4. Sharma and Pathak [22] noted that* Brassica juncea* can remediate* Pb*,* Cd,* and TPHs. About 70% of* Pb* solution of 2* mg/l* accumulated in* Brassica juncea* roots within 24 hours.* Brassica juncea* seedlings removed 40-50% of the* Cd* within 24 hours.* Cd* was removed from 20* g/l* to 9* g/l* within 24 hours [22]. Furthermore, it has been observed that* As *might be taken up by plants because of similar characteristics to the plant nutrient phosphate [22]. Rhizodegradation and humification were discovered to be the most important disappearance mechanisms for TPHs with no or little uptake [21]. Phytoremediation was able to bring TPHs levels to below the plateau level in a field contaminated with TPHs of 2000-40000* mg/kg* [22]. This shows that* Brassica juncea* performance in this study was poor as compared to other studies probably because the soil was heavily contaminated with TPHs which inhibit growth. The plant experienced stunted growth and poor germination; thus its bioremediation capabilities are limited to soil that is not heavily contaminated. This again explains why chicken droppings performed better than* Brassica juncea* in this study.

The results shown in Table 4 revealed that chicken droppings and tobacco compost were performing better in remediating soil contaminated by TPHs,* Pb*,* As,* and* Cd*. The compost performed better as compared to* Brassica juncea *probably because compost contains many different species of microorganisms that are able to adapt and metabolize pollutants. Chicken droppings as shown by Table 4 were the highest in reclaiming TPHs,* As,* and* Cd*. Tobacco compost was the highest in reclaiming* Pb*. Chicken droppings probably had high microbial biomass and species diversity which were able to degrade TPHs,* As, *and* Cd;* also tobacco compost had microbes that were able to degrade* Pb* more than chicken droppings [21]. Chicken droppings can enhance biological activity and biodegradation through the presence of NPK that enhances microbial activity [23]. This explains why chicken droppings had the highest rate of remediation in 60 days than* Brassica juncea* and tobacco compost [23]. Previous studies have also used stirred tank bioreactors stimulated with chicken droppings to treat TPHs polluted marine sediments (at a rate of 75 to 95.5% degradation of 106-116* mg/kg*) by natural microbial communities [24]. Ohiri et al. [25] observed chicken droppings rates of degradation of TPHs to be 72.18% and averages of 1.12-11.95 for* Pb*,* Cd,* and* As, *respectively. Microbial diversity and biomass differ with types of compost and tobacco compost has the little microbial diversity and hence limited survival in contaminated environments [14]. This supports the notion shown by low bioremediation rates by tobacco compost.

**Figure 3:**
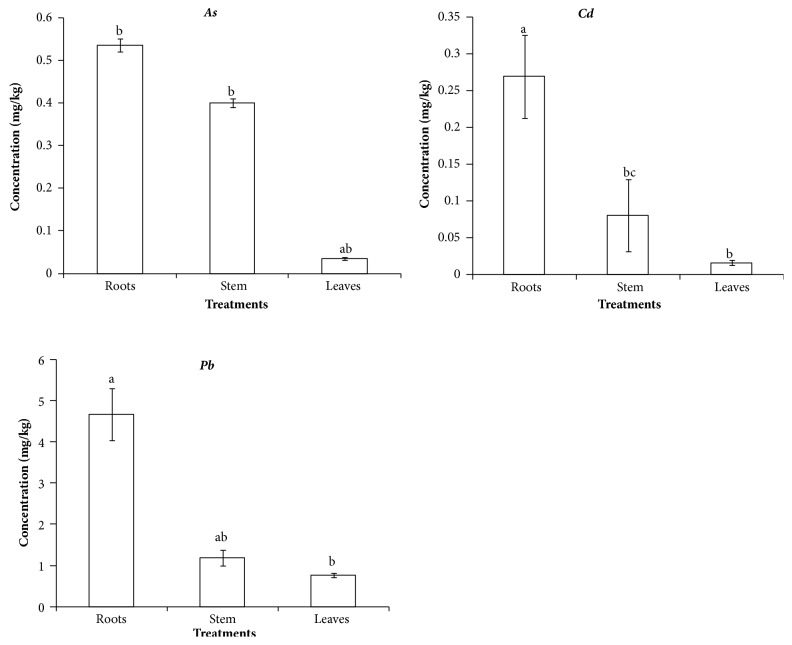
*As*,* Cd,* and* Pb* concentration level in roots, stem, and leaves.* Different superscripts indicate significant difference (p < 0.05); similar superscripts indicate no significant difference (p > 0.05). ∗SE: standard error.*

Figure 3 shows that there was high* As* concentration in the roots followed by stem and leaves. There was a significant statistical difference between roots and leaves concentrations of* As* (Figure 3). Figure 3 shows that the concentration of* Cd* was high in the roots and reduced in the stem and leaves. The roots had the highest concentration and the leaves had the lowest concentration of* Cd*. Furthermore, there was a statistically significant difference between the roots and the stem of* Cd* concentration. The general observation reveals that there was a high concentration of contaminants (*Pb, As* and* Cd*) in the roots because the roots are responsible for absorption and adsorption of soluble nutrients from the soil into the plant system [2, 4, 10]. The metals were transported from the root to the stem and then to the leaves. Thus the stem had high concentrations of metals than the leaves. This is because* Brassica juncea* grass is a hyper-accumulator and there is no clear distinction between stem and leaves. Sharma and Pathak [22] observed that* Brassica juncea* reduces leaching of metals from soil by over 98%. This is through rhizofiltration of contaminants that are soluble within the roots depth due to biotic and abiotic processes. Rhizofiltration results in the containment of contaminants through immobilization or accumulation within a plant with high concentrations in the roots, stem, and shoots [22].* Brassica juncea* removed* Pb*,* As,* and* Cd* through accumulation in their system.

## 4. Conclusion

TPHs,* Pb*,* As,* and* Cd* pollution were high at the selected sites and were above international standards (USEPA Standards 1991 and MHSPEN, 2000) maximum permissible limits. Changes in TPHs,* Pb*,* As,* and* Cd* concentrations were noted within months with the dry season month of August recording the highest. This proved the effect of meteorological conditions on TPHs,* Pb*,* As,* and* Cd* distribution and dispersion. Based on the levels of pollution at the selected site the study concluded that there is high potential of ground and surface water pollution and human health and ecological toxicity. The bioremediation pot experiment was successful in reclaiming contaminated soil with chicken droppings recording the highest remediation rate for TPHs,* As,* and* Cd* followed by tobacco compost recording the highest in reclaiming* Pb*.* Brassica juncea* was second from chicken droppings in reclaiming TPHs,* As* and* Cd*. The experiment was done for 60 days in a biostimulation ex situ experiment and it was successful.

## Figures and Tables

**Table 1:** Comparison of TPHs, *Pb*, *As,* and *Cd* concentration on the selected and control sites.

**Parameter(*mg/kg*)**	**Selected site(Mean±S.E)**	**Control site(Mean±S.E)**
TPHs	21 415±739^a^	0.06±0.03^b^
*Pb*	170.3±7.4^b^	0.87±0.60^a^
*As*	2.58±0.40^b^	0.001±0.0^a^
Cd	4.4±0.352^a^	0.001±0^b^

Different superscripts indicate significant difference (p < 0.05); similar superscripts indicate no significant difference (p > 0.05) in rows. *∗*SE: standard error.

**Table 2:** Contamination factors.

**Parameter**	**Selected Site**	**Contamination Factor Key**
TPHs	356 919	CF>6 very high contamination
*Pb*	195.1	CF>6 very high contamination
*As*	258.7	CF>6 very high contamination
*Cd*	4 400	CF>6 very high contamination

**Table 3:** Differences in TPHs, *Pb*, *As,* and *Cd* concentration levels in wet and dry season.

**Parameter *(mg/kg)***	**March**	**April**	**July**	**August**
TPHs	18701±651^b^	19624±706.611^b^	23139±521^a^	24196±459^a^
*Pb*	143.9±19.6^b^	161.6±7.06^b^	182.6±3.4^bc^	195.5±2.3^c^
*As*	1.48±0.29^b^	1.58±0.445^ab^	3.36±0.851^ac^	3.90±0.60^c^
Cd	3.6±0.57^b^	4.0±0.58^ab^	4.4±0.33^b^	5.7±0.33^ab^

Different superscripts indicate significant difference (p < 0.05). Similar superscripts indicate no significant difference (p > 0.05) in the same rows. *∗*SE: standard error.

**Table 4:** Comparison of *Brassica juncea*, tobacco compost, and chicken droppings.

**Parameter (*mg/kg*)**	***Brassica juncea***	**Tobacco Compost**	**Chicken Droppings**	**Control Experiment**
TPHs	903.7±44.4^a^	1780.9±252.8^b^	6969.8±214.7^c^	0.549±0.2^d^
*Pb*	11.88±0.45^a^	16.95±0.37^b^	13.89±0.43^c^	0.048±0.025^d^
*As*	0.389±0.436^a^	0.213±0.009^b^	0.623±0.053^c^	0.001±0^d^
Cd	0.273±0.095^b^	0.013±0.001^a^	1.277±0.155^c^	0.001±0^d^

Different superscripts indicate significant difference (p < 0.05). Similar superscripts indicate no significant difference (p > 0.05). *∗*SE: standard error.

## Data Availability

The data used to support the findings of this study are included within the article and are available from the corresponding author upon request.
